# Impact of nonclinical factors on intensive care unit admission decisions: a vignette-based randomized trial (V-TRIAGE)

**DOI:** 10.5935/0103-507X.20210029

**Published:** 2021

**Authors:** João Gabriel Rosa Ramos, Otavio Tavares Ranzani, Roger Daglius Dias, Daniel Neves Forte

**Affiliations:** 1 Clinica Florence - Salvador (BA), Brazil.; 2 Intensive Care Unit, Hospital São Rafael, Rede D’Or São Luiz - Salvador (BA), Brazil.; 3 Instituto D’Or de Pesquisa e Ensino - Salvador, Brazil.; 4 Pulmonary Division, Instituto do Coração, Hospital das Clínicas, Faculdade de Medicina, Universidade de São Paulo - São Paulo (SP), Brazil.; 5 STRATUS Center for Medical Simulation, Brigham and Women’s Hospital - Boston, MA, United States.; 6 Palliative Care Team, Hospital Sírio-Libanês - São Paulo (SP), Brazil.

**Keywords:** Critical care, Resource allocation, Decision making, Intensive care units, Cuidados críticos, Alocação de recursos, Tomada de decisões, Unidades de terapia intensiva

## Abstract

**Objective:**

To assess the impact of intensive care unit bed availability, distractors and choice framing on intensive care unit admission decisions.

**Methods:**

This study was a randomized factorial trial using patient-based vignettes. The vignettes were deemed archetypical for intensive care unit admission or refusal, as judged by a group of experts. Intensive care unit physicians were randomized to 1) an increased distraction (intervention) or a control group, 2) an intensive care unit bed scarcity or nonscarcity (availability) setting, and 3) a multiple-choice or omission (*status quo*) vignette scenario. The primary outcome was the proportion of appropriate intensive care unit allocations, defined as concordance with the allocation decision made by the group of experts.

**Results:**

We analyzed 125 physicians. Overall, distractors had no impact on the outcome; however, there was a differential drop-out rate, with fewer physicians in the intervention arm completing the questionnaire. Intensive care unit bed availability was associated with an inappropriate allocation of vignettes deemed inappropriate for intensive care unit admission (OR = 2.47; 95%CI 1.19 - 5.11) but not of vignettes appropriate for intensive care unit admission. There was a significant interaction with the presence of distractors (p = 0.007), with intensive care unit bed availability being associated with increased intensive care unit admission of vignettes inappropriate for intensive care unit admission in the distractor (intervention) arm (OR = 9.82; 95%CI 2.68 - 25.93) but not in the control group (OR = 1.02; 95%CI 0.38 - 2.72). Multiple choices were associated with increased inappropriate allocation in comparison to the omission group (OR = 5.18; 95%CI 1.37 - 19.61).

**Conclusion:**

Intensive care unit bed availability and cognitive biases were associated with inappropriate intensive care unit allocation decisions. These findings may have implications for intensive care unit admission policies.

## Abstract (pt)

**Objetivo:**

Avaliar o impacto da disponibilidade de leitos em unidade de terapia intensiva, distratores e formatação da escolha, nas decisões de admissão na unidade de terapia intensiva.

**Métodos:**

Este estudo foi um ensaio randomizado fatorial, com utilização de vinhetas baseadas em pacientes. As vinhetas foram consideradas arquetípicas para admissão ou recusa de admissão na unidade de terapia intensiva, conforme julgado por um grupo de especialistas. Médicos de unidade de terapia intensiva foram randomizados para um grupo com distrações (intervenção) ou um grupo controle; a um ambiente de escassez ou de disponibilidade de leitos em unidade de terapia intensiva (disponibilidade) e a uma vinheta com cenário de múltipla escolha ou omissão (*status quo*). O desfecho primário foi a proporção de alocações adequadas à unidade de terapia intensiva, definida como concordância com as decisões de alocação acordadas pelo grupo de especialistas.

**Resultados:**

Analisamos 125 médicos. Em termos gerais, os distratores não tiveram impacto sobre o desfecho; contudo, houve taxa diferenciada de desistências, com menos médicos no grupo intervenção tendo respondido completamente ao questionário. A disponibilidade de leitos em unidade de terapia intensiva se associou com alocações inadequadas de vinhetas consideradas não adequadas para admissão na unidade de terapia intensiva (RC = 2,47; IC95% 1,19 - 5,11), porém não com vinhetas apropriadas para admissão na unidade de terapia intensiva. Ocorreu interação significante com a presença de distratores (p = 0,007), sendo a disponibilidade de leitos na unidade de terapia intensiva associada com maior admissão na unidade de terapia intensiva de vinhetas não apropriadas para admissão na unidade de terapia intensiva no braço com distratores (intervenção) (RC = 9,82; IC95% 2,68 - 25,93), porém não no grupo controle (RC = 5,18; IC95% 1,37 - 19,61).

**Conclusão:**

A disponibilidade de leitos em unidade de terapia intensiva e vieses cognitivos se associaram com decisões inadequadas de alocação à unidade de terapia intensiva. Esses achados podem ter implicações para políticas de admissão na unidade de terapia intensiva.

## INTRODUCTION

Intensive care unit (ICU) admission triage is performed routinely worldwide,^(1)^ and ICU refusal may be associated with worse outcomes.^(2,3)^ It has been shown that this triage process is associated with the patients’ clinical characteristics^(2,4,5)^ but is also influenced by nonclinical factors, such as ICU bed availability.^(2,5-7)^ Moreover, concern has been raised that these clinical judgments could mask prejudice or bias.^(1,7)^

Intensive care unit bed scarcity has been associated with increased ICU refusal rates,^(3,5)^ but its impact on hospital mortality or other processes of care is controversial.^(8,9)^ In some settings, ICU bed scarcity has been linked to changes in goals-of-care decisions,^(9)^ with a lower probability of ICU admission and no impact on mortality, leading to questioning of the appropriateness of those admission processes.^(1,10)^

Specifically, ICU bed scarcity could lead to unintended ICU refusal due to the absence of available beds for admission, which could impact patient outcomes. However, the opposite could also be true, and increased availability of ICU beds could lead to increased admission of patients unlikely to benefit from intensive care. Alternatively, reduced ICU bed availability might be a surrogate of an increased strain, which could be associated with increased distractors, more pronounced influences on cognitive biases and inappropriate decision-making, leading to potentially inappropriate ICU admissions or refusals.^(11)^

In health care, it has been suggested that the presence of cognitive biases^(12,13)^ is associated with poor decision-making. For example, the frame in which choices are presented may lead to different decisions, and the term “*status quo*” bias (or omission bias) has been described as the “people’s tendency to maintain one’s current or previous decision”,^(14)^ while “multiple-choice” bias has been used to describe situations in which “multiple options can paradoxically influence people to choose an option that would have been declined if fewer options were available”,^(13)^ and both biases have been shown to influence medical decision-making.^(12,13)^

Ameliorating ICU allocation processes may improve patient outcomes and resource consumption. To characterize the decision-making processes associated with ICU admission, we sought to evaluate the impact of ICU bed availability and distractors and of framing choices on ICU admission decisions.

## METHODS

This study was approved by the *Hospital das Clínicas* of the *Faculdade de Medicina* of the *Universidade de São Paulo* (USP) institutional review board (approval number 1.015.441), which approved the utilization of electronically obtained informed consent. The study protocol was registered at clinicaltrials.gov (NCT02430454).

This study was an open-label, vignette-based randomized trial with a factorial design. All respondents were requested to complete a demographic questionnaire and to respond to six clinical vignettes, as described below. The order of the vignettes and the order of the alternative responses in each vignette were randomly presented to avoid any carry-over effect, framing or availability biases induced by the questionnaire. Simple randomization was performed using SurveyMonkey® (SurveyMonkey Inc., USA) web-based software.

All participants were submitted to three sets of randomizations to test three distinct hypotheses. In the first randomization set, all respondents were randomized either to a distractor or a control group. In the second randomization set, all respondents were randomized to different versions of clinical vignettes. Five vignette scenarios were presented, either as ICU bed scarcity or availability versions. Each respondent was randomized to one of two groups in each vignette scenario, so all participants responded to all clinical vignettes. In the third randomization set, all respondents were randomized to respond to a vignette either in a multiple-choice, active decision scenario or in an omission (*status quo*) scenario.

### Distractors versus control groups

The first hypothesis was that visual and auditory distractors were associated with inappropriate ICU allocation. To test this hypothesis, respondents were randomized to either the distractors or the control group. Allocation was initially defined as 1:1; however, a preplanned interim analysis, with 70% of the expected sample size, found that there was a differential dropout rate, with a lower response rate in the distractor group, so it was decided to change the allocation to 2:1 with more respondents randomized to the distractor group.

After logging in to the website, respondents in the distractor group were instructed to answer the questions according to their intuition as quickly as possible. They were also presented with distracting videos and sounds and a 3-minute alarm timer in each vignette. To avoid reflection about the topic, those respondents who had access to the demographic questionnaire only had access to it after answering the vignettes questions.

Respondents in the control group were instructed to answer the questions in a calm and thoughtful manner without any time limitations. There were no intentional distractions, and those respondents were exposed to the demographic questionnaire before answering the vignettes.

### Intensive care unit scarcity or availability groups

The second hypothesis was that ICU bed scarcity would be associated with inappropriate refusal of patients deemed appropriate for ICU admission, while ICU bed availability would be associated with inappropriate admission of patients deemed inappropriate for ICU admission. This hypothesis was tested with clinical vignettes archetypical for ICU admission or refusal and nonarchetypical clinical vignettes, both as single or as multiple vignettes, because the representativeness of the cases has been shown to influence decision-making.^(15)^

Five groups of vignettes were built, and each vignette group was randomized independently, so each respondent could be exposed to vignettes in both ICU scarcity and ICU availability settings (Appendix 1S and Table 1S - Supplementary material). Allocation was defined as 1:1 for each of these randomizations. Appropriateness of the ICU allocation was defined as an allocation that was concordant with the archetypical status of the vignette scenario (archetypical for ICU admission or refusal).

Group A (single vignette archetypical for admission) comprised a single vignette archetypical for ICU admission, randomly presented in two scenarios: an ICU bed availability setting (three available ICU beds) or an ICU scarcity setting (last bed scenario). The respondent was to determine whether the patient in each vignette scenario should be admitted or refused ICU admission. Appropriateness of the allocation was defined as ICU admission.

Group B (multiple vignettes archetypical for admission) contained two vignettes archetypical for ICU admission, randomly presented in two scenarios: an ICU bed availability setting (three available ICU beds) or an ICU scarcity setting (the last two beds scenario). The respondent was to determine if both cases should be admitted, if only one of them should be admitted or if both should be refused ICU admission. The appropriateness of the allocation was defined as ICU admission of both cases.

Group C (single vignette archetypical for refusal) comprised a single vignette archetypical for ICU refusal, randomly presented in two scenarios: an ICU bed availability setting (three available ICU beds) or an ICU scarcity setting (last bed scenario). The respondent needed to determine whether the patient in the vignette scenario should be admitted or refused ICU admission. The appropriateness of the allocation was defined as ICU refusal.

Group D (multiple vignettes archetypical for refusal) contained two vignettes archetypical for ICU refusal, randomly presented in two scenarios: an ICU bed availability setting (three available ICU beds) or an ICU scarcity setting (last two beds scenario). It was required that the respondent determined if both cases should be admitted, if only one of them should be admitted or if both should refuse ICU admission. The appropriateness of the allocation was defined as ICU refusal of both cases.

Group E (nonarchetypical vignette) comprised a single vignette, randomly presented in two scenarios: an ICU bed availability setting (three available ICU beds) or an ICU scarcity setting (last bed scenario). This single vignette was not archetypical for ICU refusal or admission, so there was no *a priori* defined appropriateness of allocation.

### Multiple-choice decision or omission (status quo) groups

Because the constraint of choosing between more than one patient to be admitted to the ICU could lead to inappropriate ICU allocation, it was hypothesized that presenting multiple choices could impact the decision-making process. To test this third hypothesis, group F (cognitive bias vignettes) respondents were randomized to either a multiple-choice decision group or an omission (*status quo*) group. Allocation was defined as 1:1 for each of these randomizations.

In the multiple-choice decision version, respondents were presented with the active decision to admit to the last ICU bed either an urgent case archetypical for ICU admission or to reserve this same last ICU bed for an asymptomatic patient who would be subjected to elective abdominal aortic aneurysm repair. The appropriate allocation decision would be to admit the urgent case archetypical for ICU admission.

In the omission (*status quo*) version, the last ICU bed was already reserved for the asymptomatic patient who would be subjected to elective abdominal aortic aneurysm repair, and the respondents were presented with the decision of maintaining the bed reservation (omission) or to cancel the reservation and admit one of two different urgent cases archetypical for ICU admission. The appropriate allocation decision would be to admit one of the two archetypical cases and cancel the surgery reservation.

### Demographic questionnaire

The online research questionnaire included demographic and professional characteristics of the respondents and their ICUs, such as whether there was high-intensity staffing (defined as the presence of a critical care specialist at least 4 hours per day, at least 5 days per week) and variables related to the respondents’ exposure to situations of ICU bed scarcity and triage in their practice.

### Vignettes development

Clinical vignettes were developed based on representative real patients for whom ICU admission was requested at HCFMUSP in January 2014. Those vignettes contained information such as age, sex, length of hospitalization, comorbidities, previous functional status, acute diagnosis, the presence of organ dysfunctions, the need for advanced life support and an objective reason for ICU admission request (Appendix 1S - Supplementary material).

The vignettes were tested for concordance with ICU admission or refusal by eight physicians with experience in critical care or emergency medicine. Those physicians evaluated the appropriateness of ICU admission for each vignette. Vignettes were considered archetypical for ICU admission (appropriate admission) or archetypical for ICU refusal (inappropriate admission) if more than 80% of the physicians agreed on ICU allocation in an ICU scarcity setting (Table 1S - Supplementary material).

### Participants

A convenience sample of Brazilian physicians with experience in critical care was invited to participate in the study by medical specialty e-mail groups, social media networking and personal contacts. Invitations were sent on three different occasions at 2-week intervals beginning in October 2015.

Respondents were included if they were licensed practicing physicians currently working in ICUs. Participants were excluded if consent was not obtained or if the research questionnaire was not fully completed, since otherwise it would not be possible to track if the respondent was actually a licensed physician and whether the response was a duplicate. It was not required to be board certified in critical care to participate in this study.

### Outcomes

The primary outcome was defined as the proportion of scenarios with appropriate ICU allocation in each vignette group. Perceived difficulty answering each question was defined as a secondary outcome of interest. Difficulty in answering each question was evaluated on a Likert scale from 1 to 5, with 5 representing a very difficult question.

Surrogate markers, such as the time to complete the questionnaire and any perceived difficulty answering the questions, were used to assess the impact of the intervention.

### Statistical analysis

The sample size was calculated as 104 respondents to detect any effect of each intervention on the primary outcome, considering an appropriate ICU allocation of 80% in the control group and 54% in the experimental group, a study power of 80%, and a significance threshold of 0.05. Those effect sizes were based on the effect of the cognitive load taken from previously published data on trauma triage^(15)^ and were considered to be similar for each intervention. Anticipating a complete response rate of 60%, 174 participants would have to be randomized. The complete response rate was defined as the proportion of respondents who logged in and completed the questionnaire. Only respondents with complete responses were analyzed.

Microsoft Excel 365 (Microsoft, United States) and Statistical Package for Social Science (SPSS), version 13.0 (SPSS Inc., United States) were utilized as databases and statistical software.

Continuous data are described as the mean ± standard deviation or median (interquartile range) and were analyzed by analysis of variance (Anova) or the Mann-Whitney U test as appropriate. Categorical variables were described as numbers (percentages) and were analyzed by Chi-square tests, Fisher’s exact test or McNemar’s test, as appropriate. Secondary analyses were performed to assess the interaction between the interventions. Since the power to detect significant interactions in this study may be limited, it was decided to present the results both by the trial factor (interventions compared separately) and by the interaction group. Because all analyses were prespecified and were presented in the manuscript (irrespective of whether they were statistically significant), we did not perform any adjustment for multiple comparisons.^(16)^ A two-tailed p-value of 0.05 was considered significant in all analyses.

## RESULTS

**Figure 1:**
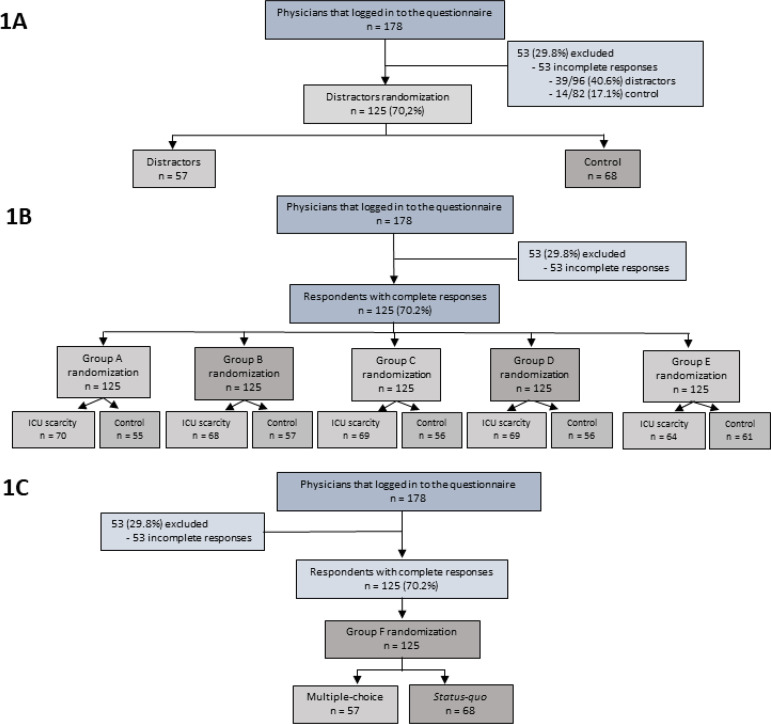
Study enrollment and randomization flow diagram, according to distractor (A) or ICU scarcity (B) or multiple-choice/*status quo* (C) randomizations. ICU - intensive care unit.

From October 2015 to December 2015, 178 physicians logged into the electronic questionnaire, and 125 (70.2%) had complete responses and their data were analyzed (Figure 1). Each physician completed 6 different vignette scenarios for a total of 750 scenarios. The physicians practiced medicine in 15 different Brazilian states; their mean age was 37.4 ± 7.3 years, 96 (76%) were board certified in critical care, and approximately 30% were rarely or never exposed to situations of ICU bed scarcity.

Overall, 96 respondents (53.9%) were randomized to the distractor group, and 82 respondents (46.1%) were randomized to the control group. There was a difference in the completion rate between the distractors and control groups (Figure 1A). Fifty-seven (59.4%) respondents completed the questionnaire in the distractor group, and 68 (82.9%) respondents completed the questionnaire in the control group (p = 0.001). There was no indication of systematic differences in the primary outcome when comparing available responses of physicians with complete or incomplete responses (Table 2S - Supplementary material).

### Distractors or control groups

**Figure 2:**
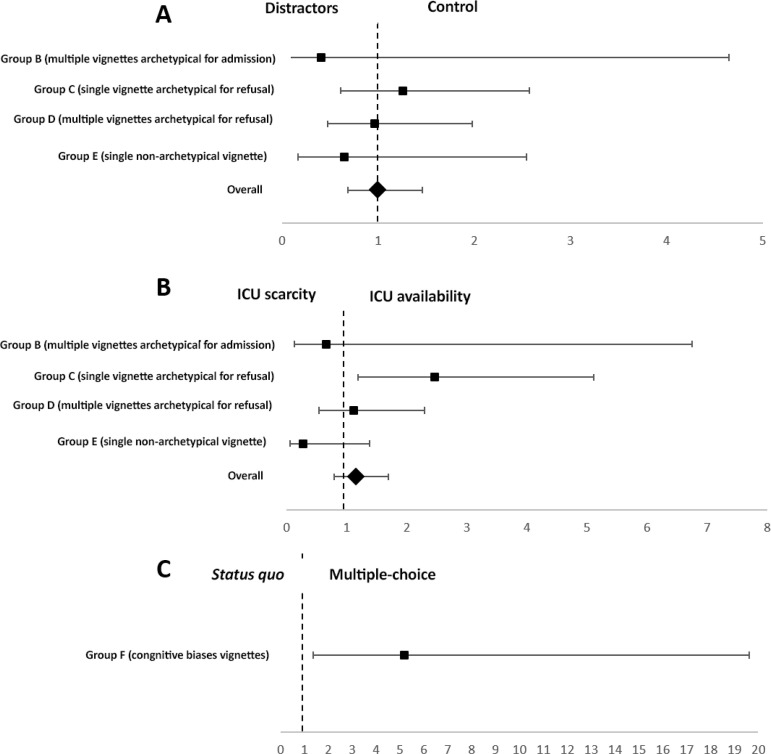
Impact on inappropriate intensive care unit allocation of case vignettes according to (A) distractor randomization, (B) intensive care unit scarcity randomization and (C) multiple-choice/*status quo* randomization. Group A does not appear in the figure because there was no inappropriate allocation in any randomization groups. ICU - intensive care unit.

There were no differences in the baseline characteristics between the distractors and the control groups (Table 1). Overall, when analyzing all 750 scenarios combined, there were no differences among the randomization groups because inappropriate allocation occurred in 19.6% (67/342) of the scenarios under increased distractors and in 20.8% (85/408) of the scenarios in the control group, p = 0.673. When analyzing ICU allocation in each vignette group, there was no difference in the primary outcome between the distractors and control groups (Figure 2A and Table 3S - Supplementary material). Moreover, the time to complete the questionnaire and the perceived difficulty in answering the questions were not different between the distractor and control groups (Table 1).

**Table 1:** Baseline characteristics according to the distractors or the control randomization group

Characteristic	Control (n = 68)	Distractors (n = 57)	p value
Time to complete questionnaire (minutes)	21.4 ± 22.1	19.4 ± 31.9	0.677
Age	37.1 ± 6.3	37.7 ± 8.4	0.677
Male sex	43 (64.2)	44 (77.2)	0.121
Years of medical practice	12.6 ± 6.9	13.3 ± 8.9	0.584
Board certified in critical care	54 (79.4)	41 (71.9)	0.402
Average hours working in ICU per week (hours)			0.303
< 12	2 (2.9)	2 (3.5)	
12 - 24	5 (7.4)	10 (17.5)	
24 - 40	19 (27.9)	17 (29.8)	
> 40	42 (61.8)	28 (49.1)	
"Closed" ICU	38 (55.9)	39 (68.4)	0.151
Public ICU	34 (50)	24 (42.1)	0.378
High-intensity staff ICU	66 (97.1)	57 (100)	0.192
Number of ICU beds	24 ± 17	20 ± 14	0.201
Experience of situations of ICU beds scarcity			0.992
Never	3 (4.4)	3 (5.3)	
Rarely	18 (26.5)	14 (24.6)	
Sometimes	17 (25)	16 (28.1)	
Frequently	17 (25)	13 (22.8)	
Always	13 (19.1)	11 (19.3)	
Involved in ICU triage			0.332
Never	20 (29.4)	11 (19.3)	
Rarely	9 (13.2)	12 (21.1)	
Sometimes	14 (20.6)	11 (19.3)	
Frequently	19 (27.9)	13 (22.8)	
Always	6 (8.8)	10 (17.5)	
Previous training in ICU triage	12 (17.6)	8 (14)	0.583
Perceived difficult in answering the complete questionnaire	2.5 (2.0 - 3.0)	2.0 (2.0 - 3.0)	0.469

ICU - intensive care unit. Results are expressed as the mean ± standard deviation, n (%) or median (interquartile range).

### Intensive care unit scarcity or availability vignettes

There was no significant imbalance between the characteristics of the respondents among the randomized vignette groups (Table 4S - Supplementary material). Figure 2B demonstrates the impact of randomization on the proportion of cases admitted or refused ICU admission in each vignette group and the perceived difficulty in responding to each vignette. Cases deemed appropriate for ICU admission (Groups A and B) were almost universally admitted to the ICU, regardless of ICU bed availability (Figure 2B and Table 5S - Supplementary material). Group E vignettes (nonarchetypical vignettes) were also generally admitted to the ICU in both randomization groups, even though there was a trend toward increased refusal in the ICU scarcity setting (3.3% *versus* 10.9%; odds ratio - OR = 3.62; 95% confidence interval - 95%CI 0.72 - 18.18).

Cases deemed inappropriate for ICU admission (Groups C and D) were admitted to the ICU in 52 (41.6%) and 74 (59.2%) of the case scenarios, respectively. Group C vignettes (archetypical refusal case) were more often inappropriately admitted in the ICU bed availability setting than in the ICU scarcity setting (OR = 2.47; 95%CI 1.19 - 5.11), as seen in figure 2B and table 5S (Supplementary material).

### Multiple-choice decision and omission (status quo) vignettes

There was no difference in the baseline characteristics between the groups of respondents (Table 6S - Supplementary material). The respondents were more prone to choose an inappropriate allocation option (i.e., admit the elective surgery patient instead of a patient archetypical for ICU admission) in the multiple-choice decision group than in the omission (*status quo*) group (OR = 5.18; 95%CI 1.37 - 19.62) (Figure 2C and Table 7S - Supplementary material).

### Perceived difficulty in answering the vignettes

**Figure 3:**
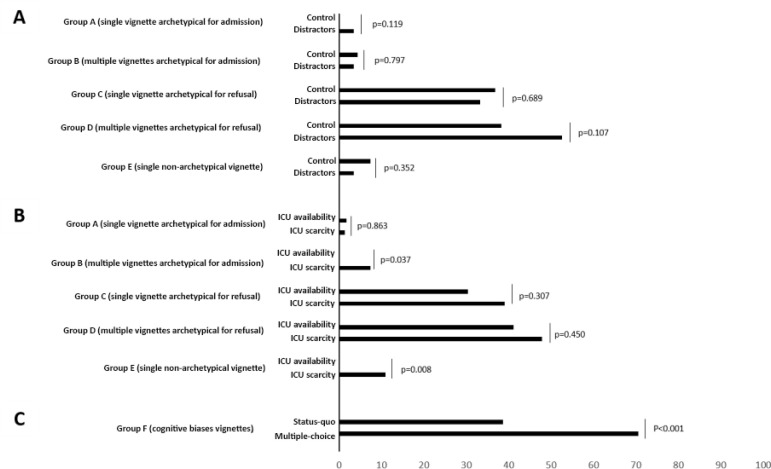
Percentage of respondents rating questions as difficult in the (A) distractor randomization, (B) intensive care unit scarcity randomization and (C) multiple-choice/*status quo* randomization groups. ICU - intensive care unit.

Allocation decisions regarding archetypical case scenarios for ICU refusal were perceived as more difficult than those regarding archetypical case scenarios for ICU admission. Multiple-choice decision and omission (*status quo*) (Group F) vignettes were rated as the most difficult to answer (Figure 3, Table 5S and Table 7S - Supplementary material).

### Analysis of interactions

The analysis of interactions is shown in table 2, and the total number of patients in each group of interactions is depicted in the supplementary material (Table 8S - Supplementary material). There was a significant interaction between distractors and ICU scarcity only in Group C (p = 0.007). Analysis of the interaction demonstrated that patients in Group C were more often admitted to the ICU (inappropriate allocation) in the ICU availability setting (compared to ICU scarcity) in the distractor group (OR = 9.82; 95%CI 2.68 - 25.93) but not in the control group (OR = 1.02; 95%CI 0.38 - 2.72). No effect of interactions was apparent between the distractors and the other vignette groups.

**Table 2:** Inappropriate allocations in each randomization group and analysis of interactions

	Control	Distractors	p value	Interaction between vignette group and cognitive load
Group A				
Control	0 (0)	0 (0)	NA	NA
ICU scarcity	0 (0)	0 (0)	NA	
Group B				
Control	0 (0)	1 (3.7)	0.288	0.592
ICU scarcity	1 (1)	1 (3.3)	0.865	
Group C				
Control	12 (44.4)	18 (62.1)	0.186	0.007
ICU scarcity	18 (43.9)	4 (14.3)	0.010	
Group D				
Control	18 (60)	16 (61.5)	0.906	0.94
ICU scarcity	22 (57.9)	18 (58.1)	0.989	
Group E				
Control	1 (3.2)	1 (3.3)	0.981	0.48
ICU scarcity	3 (8.1)	4 (14.8)	0.396	
Group F				
Multiple-choice	7 (22.6)	4 (15.4)	0.493	0.935
*Status-quo*	3 (8.1)	0 (0)	0.105	

ICU - intensive care unit; NA - not applicable. Results are expressed as n (%).

## DISCUSSION

In this sample of Brazilian physicians with experience in critical care, the presence of distractors did not demonstrate any direct effect on ICU admission decisions, but these results may have been hampered by the differential dropout ratio. ICU bed availability was associated with increased ICU admission in case scenarios deemed inappropriate for ICU admission but had no impact on scenarios deemed appropriate for ICU admission. However, this effect was subjected to interactions with distractors. Moreover, the active choice between case scenarios competing for ICU admission led to more inappropriate refusals of admission than the alternative version of inappropriate admission.

The dual-process model of cognitive reasoning postulates that intuitive judgment systems work in two ways: while system 2 is an analytical system operating on rule-derived deduction, system 1 is an automatic, pattern-recognition system based on the use of heuristics.^(17)^ Increased use of system 1, with pitfalls associated with a higher chance of biases, depends on characteristics of the task, such as the cognitive load, and of the individual performing the task, such as the degree of expertise.^(17)^

In this study, contrary to previous psychological^(18,19)^ and health care research,^(15,20)^ the presence of distractors had no impact on decisions made by the respondents. Distractors may lead to increased cognitive load, which has been shown to augment the utilization of heuristics, which can lead to poor decision-making.^(15,18,19)^ Cognitive load is difficult to directly measure,^(21)^ so surrogate markers, such as subjective mental effort rates, time efficiency measures and task performance measures, may be used instead.^(15,22)^ We found no difference in the time to complete the questionnaire or in the perceived difficulty in responding to the vignettes when comparing distractors and control groups. It is possible, then, that the methods chosen were not effective. Alternatively, the differential completion rate between the distractors and control groups may indicate that this randomization was subject to bias. Even though we found no differences when analyzing the responses of physicians with complete and incomplete responses, it is not possible to exclude that resilience, or other unmeasured factors, may have affected the results. Another hypothesis is that the other groups of randomization *per se* could have had an effect on cognitive load, modifying the effect of distractor randomization.

Intensive care unit bed availability was associated with increased admission of case scenarios deemed inappropriate for ICU admission but had no impact on scenarios deemed appropriate for ICU admission or nonarchetypical cases. The absence of an impact of ICU scarcity on the admission of appropriate cases was expected and is consistent with the notion of physicians’ advocacy for individual patients, even in the presence of limited resources. There was no statistically significant impact of ICU scarcity on the nonarchetypical vignette group (Group C), but there was an absolute difference of 7.6% of refusal and a greater perceived difficulty in answering the question in the scarcity version, which may reflect a greater dilemma about resource allocation in this more borderline group.

This study demonstrated that cases deemed inappropriate for ICU admission would often be admitted to the ICU and that this admission decision may be dependent on ICU bed availability. The “rule of rescue”,^(23,24)^ or the fact that individual physicians may devote substantial resources to patients unlikely to benefit from them, is a pattern of behavior previously observed in critical care settings.^(23)^ This pattern of admission has also been demonstrated by cohort studies evaluating ICU admissions with and without ICU bed constraints, which have suggested that a greater availability of ICU beds may lead to inappropriate ICU admissions.^(9,10)^ The fact that respondents rated the vignettes associated with cases deemed inappropriate for ICU admission as more difficult may reflect the dilemma between desiring care for the individual patient and resource constraints or, alternatively, the dilemma associated with what clinicians think they should do and what they would actually do.^(25)^ Moreover, it was demonstrated that there was an interaction between ICU scarcity and distractors, with the distractor intervention leading to increased inappropriate allocation of patients archetypical for ICU refusal. This may suggest that some of those decisions are more prone to judgment errors when the physicians are placed under an increased cognitive load.

This study has specifically addressed the impact of active, multiple-choice decisions *versus* omissions (*status quo*) on triage decisions because physicians are often confronted with the decision not only to choose if one patient should be admitted to the ICU but also to choose which patient, among others, should be admitted to the ICU. It has been previously suggested that even highly trained individuals such as physicians, when confronted with complex decisions, usually prefer inaction that preserves the *status quo* than action that changes it, even when this change may lead to the best outcomes,^(12)^ and that these biases are more pronounced when more options are presented.^(13)^

Those assumptions were tested by presenting a “dummy” case (an elective surgery patient) among cases archetypical for ICU admission in two versions. The “dummy” case was presented as either as an active choice between the admission of the “dummy” or an archetypical case or as a choice between preserving the *status quo* and maintaining the reservation for admission of the “dummy” case or changing it through the action of admitting one archetypical case. We found little evidence of omission bias, since only a small percentage of respondents would maintain the reservation for the elective surgical case. However, when confronted with the decision to actively choose between cases, even when there should be a clear priority, physicians often chose an inappropriate allocation option. The difficulty in performing such a choice is further supported by the fact that the response to this group was rated as the most difficult.

This randomized trial is one of the few studies evaluating cognitive factors associated with ICU admission decisions^(7)^ and, to our knowledge, is the first to specifically address the impact of distractors, ICU bed availability, and specific cognitive biases. However, despite utilizing methods similar to what had already been utilized in the literature,^(7,15,26)^ this study was not able to find an objective effect of distractors on surrogate markers of cognitive load. Even though there was probably an effect, as there was a differential dropout among the randomization groups, it is not possible to infer the direction of this effect, making it difficult to interpret these findings. However, this does not invalidate the results associated with the other factorial randomizations (i.e., ICU bed availability and active/omission randomizations). Those limitations are especially important because factorial trials may be efficient tools for analyzing more than one intervention simultaneously within a smaller sample size than would be required to perform standard parallel clinical trials.^(27)^ However, this sample size assumption is based on the premise that there should be no interaction between treatment arms.^(28)^ When this assumption is not met, it is likely that the trial may be underpowered to detect significant interactions. Although this is the case in our trial and the power to detect significant interactions in this study may be limited because the primary comparisons were the main effects sought, our approach to analyzing and reporting the data may be justifiable.^(28,29)^ Moreover, we performed simple randomization, which, due to the small sample size, may have led to unmeasured imbalances in the study groups, even though no clinically important differences were detected among the groups. Another limitation is that although the respondents had a wide distribution among 15 different Brazilian states, this study was derived from a convenience sample of Brazilian physicians, which may limit the generalizability of our results.

Moreover, the clinical vignette methodology is based on static descriptions, subjected to different interpretations, and may be less robust than encounters with real patients. Vignettes offer significant advantages, such as quantification of physician performance, ease of use and low cost,^(30,31)^ and as such, several studies have used case vignettes to study clinicians’ attitudes. However, the vignette methodology differs from real patients in important ways because it summarizes and standardizes important clinical information. Even though most evidence supporting the validity of vignettes to study physician behavior comes from low-risk conditions,^(12,13,31-33)^ vignettes have also been used to study clinicians’ attitudes in acute, time-sensitive settings, including the triage of critically ill patients.^(7,15,30,34-37)^ The limitations associated with the clinical vignette methodology were addressed in this study by utilizing electronic vignettes with embedded multimedia as the methodological instrument in an attempt to modulate external factors that could impact the decision-making process, such as time pressure and cognitive load.^(30)^ Moreover, these vignettes were constructed based on real patients and were validated by a group of experts, which should establish a surrogate gold standard for appropriateness for ICU admission.^(37)^ Additionally, different types of vignettes were used, evaluating clinical vignettes considered to be archetypical for ICU admission, archetypical for ICU refusal and nonarchetypical cases. It was hypothesized that respondents would make different decisions under the intervention strategies for each different type of vignette. Furthermore, it may not be feasible to conduct such a study utilizing real patients, and alternatives, such as serious game technology,^(15)^ have been proposed but have not been widely tested or are not widely available.

These findings may have implications for the development of ICU admission policies because it is likely that several external and clinician-associated factors are associated with the decision to admit a patient to the ICU. These decision-making processes should be addressed by institutional and society guidelines and possibly enhanced through the utilization of decision-aid instruments.

## CONCLUSION

In this randomized vignette-based trial, the presence of distractors had no impact on intensive care unit admission decisions, but these results may have been hampered by a differential dropout ratio. Intensive care unit bed availability was associated with increased admission of case scenarios for whom intensive care unit admission was deemed inappropriate. However, this effect was subjected to interactions with distractors. Furthermore, active decisions had more impact than omission bias in inappropriate intensive care unit allocation. These findings may have implications for the development of intensive care unit admission policies.
