# Supplementary material for: Impact of nonclinical factors on intensive care unit admission decisions: a vignette-based randomized trial (V-TRIAGE)
Source: Rev Bras Ter Intensiva. 2021 Apr-Jun;33(2):219–30. doi: 10.5935/0103-507X.20210029 (PMC8275078; doi:10.5935/0103-507X.20210029)
Supplement: Supplementary file 1 [file rbti-33-02-0219-suppl01.pdf]

Impact of non-clinical factors on intensive care unit admission decisions: a vignette-based randomized trial (V-TRIAGE)

Impacto de fatores não clínicos nas decisões relacionadas à admissão em unidade de terapia intensiva: um ensaio randomizado com base em vinhetas (V-TRIAGE)

João Gabriel Rosa Ramos[1,2,3], Otavio Tavares Ranzani[4], Roger Daglius Dias[5], Daniel Neves Forte[6]

APPENDIX 1S - CLINICAL VIGNETTES EVALUATED BY RESPONDENTS

Group A - Single vignette archetypical for intensive care unit admission

A.1. Non-intensive care unit bed scarcity setting

You are the intensivist in charge of the intensive care unit (ICU) and is also responsible for decisions of ICU admission, when there is a request for the following patient. At the moment, there are three available beds for admission and no other admission request.

Female patient, 37 years-old, admitted to the hospital three days ago. Previous history of chronic kidney disease for 12 years. She was submitted to a kidney transplant (dead donor) 3 days ago and is still in need of renal replacement therapy in the second post-operative day. Functionally independent for activities of daily living. Today, during dialysis, she developed sudden hypotension, associated with hemorrhagic drainage from surgical drains. Surgical team is aware and in the way. ICU admission was requested for somnolence, shock in use of vasopressors and need for urgent hemodialysis and surgical evaluation.

Considering the situation described above and the patient described on the vignette, what is your decision regarding ICU admission of this patient?

a) Would admit the patient
b) Would refusal admission of the patient

A.2. Intensive care unit bed scarcity setting

You are the intensivist in charge of the intensive care unit (ICU) and is also responsible for decisions of ICU admission, when there is a request for the following patient. At the moment, there is only one available bed for admission, there is no other patient in conditions of discharge from the ICU and there are scheduled surgeries for the following day.

Female patient, 37 years-old, admitted to the hospital three days ago. Previous history of chronic kidney disease for 12 years. She was submitted to a kidney transplant (dead donor) 3 days ago and is still in need of renal replacement therapy in the second post-operative day. Functionally independent for activities of daily living. Today, during dialysis, she developed sudden hypotension, associated with hemorrhagic drainage from surgical drains. Surgical team is aware and in the way. ICU admission was requested for somnolence, shock in use of vasopressors and need for urgent hemodialysis and surgical evaluation.

Considering the situation described above and the patient described on the vignette, what is your decision regarding ICU admission of this patient?

a) Would admit the patient
b) Would refusal admission of the patient

Group B - Two vignettes archetypical for intensive care unit admission

B.1. Non-ICU bed scarcity setting

You are the intensivist in charge of the intensive care unit (ICU) and is also responsible for decisions of ICU admission, when there is a request for the following patient. At the moment, there are three available beds for admission and no other admission request.

Patient 1. Male patient, 66 years-old, admitted to the hospital hours ago. Previous history of hypertension, dyslipidemia, obesity, diabetes, atrial fibrillation, ischemic stroke 5 years ago with visual deficit. Functionally independent for activities of daily living. He was admitted with a history of cough, breathless and fever that had begun 6 days before admission. He developed respiratory distress and hypotension, with need for orotracheal intubation and vasopressors. ICU admission was requested for septic shock, with the need for vasoactive drugs and invasive mechanical ventilation.

Patient 2. Male patient, 54 years-old, admitted to the hospital hours ago. Previous history of alcohol abuse. Functionally independent for activities of daily living. He was admitted for decreased level of consciousness, with history of seizures and fever that had begun three days before admission. He was intubated and submitted to a head computerized tomography (that did not show alterations) and a study or cerebrospinal fluid that was compatible with bacterial meningitis. ICU admission was requested for severe meningitis with the need for invasive mechanical ventilation.

Considering the situation described above and the patients described on the vignettes, what is your decision regarding ICU admission of these patients?

a) Would admit patient 1 and refuse admission of patient 2
b) Would admit patient 2 and refuse admission of patient 1
c) Would refuse admission of both patients (1 and 2)
d) Would admit both patients (1 and 2)

B.2. ICU bed scarcity setting

You are the intensivist in charge of the intensive care unit (ICU) and is also responsible for decisions of ICU admission, when there is a request for the following patient. At the moment, there are only two available beds for admission, there is no other patient in conditions of discharge from the ICU and there are scheduled surgeries for the following day.

Patient 1. Male patient, 66 years-old, admitted to the hospital hours ago. Previous history of hypertension, dyslipidemia, obesity, diabetes, atrial fibrillation, ischemic stroke 5 years ago with visual deficit. Functionally independent for activities of daily living. He was admitted with a history of cough, breathless and fever that had begun 6 days before admission. He developed respiratory distress and hypotension, with need for orotracheal intubation and vasopressors. ICU admission was requested for septic shock, with the need for vasoactive drugs and invasive mechanical ventilation.

Patient 2. Male patient, 54 years-old, admitted to the hospital hours ago. Previous history of alcohol abuse. Functionally independent for activities of daily living. He was admitted for decreased level of consciousness, with history of seizures and fever that had begun three days before admission. He was intubated and submitted to a head computerized tomography (that did not show alterations) and a study or cerebrospinal fluid that was compatible with bacterial meningitis. ICU admission was requested for severe meningitis with the need for invasive mechanical ventilation.

Considering the situation described above and the patients described on the vignettes, what is your decision regarding ICU admission of these patients?

a) Would admit patient 1 and refuse admission of patient 2
b) Would admit patient 2 and refuse admission of patient 1
c) Would refuse admission of both patients (1 and 2)
d) Would admit both patients (1 and 2)

Group C - Single vignette archetypical for intensive care unit refusal

C.1. Non-intensive care unit bed scarcity setting

You are the intensivist in charge of the intensive care unit (ICU) and is also responsible for decisions for ICU admission, when there is a request for the following patient. At the moment, there are three available beds for admission and no other admission request.

Female patient, 68 years-old, admitted to the hospital two weeks ago. Previous history of bladder cancer with cystectomy 5 years ago and advanced Alzheimer's disease. Completely dependent for activities of daily living. She was admitted in the hospital for treatment of an aspirative pneumonia and is evolving with worsening of the infection and has developed non-oliguric acute kidney injury and worsening of somnolence. ICU admission was requested for neurological and respiratory monitoring, without need for artificial life support at this moment.

Considering the situation described above and the patient described on the vignette, what is your decision regarding ICU admission of this patient?

a) Would admit the patient
b) Would refusal admission of the patient

C.2. Intensive care unit bed scarcity setting

You are the intensivist in charge of the intensive care unit (ICU) and is also responsible for decisions of ICU admission, when there is a request for the following patient. At the moment, there is only one available bed for admission, there is no other patient in conditions of discharge from the ICU and there are scheduled surgeries for the following day.

Female patient, 68 years-old, admitted to the hospital two weeks ago. Previous history of bladder cancer with cystectomy 5 years ago and advanced Alzheimer's disease. Completely dependent for activities of daily living. She was admitted in the hospital for treatment of an aspirative pneumonia and is evolving with worsening of the infection and has developed non-oliguric acute kidney injury and worsening of somnolence. ICU admission was requested for neurological and respiratory monitoring, with no need for artificial life support at this moment.

Considering the situation described above and the patient described on the vignette, what is your decision regarding ICU admission of this patient?

a) Would admit the patient
b) Would refusal admission of the patient

Group D – Two vignettes archetypical for intensive care unit refusal

D.1. Non-intensive care unit bed scarcity

You are the intensivist in charge of the intensive care unit (ICU) and is also responsible for decisions of ICU admission, when there is a request for the following patient. At the moment, there are three available beds for admission and no other admission request.

Patient 1. Female patient, 70 years-old, admitted to the hospital 22 days ago. Previous history of severe neurological sequelae due to stiff-person syndrome, refractory to treatments, already tracheostomized and with planned gastrostomy. Completely dependent for activities of daily living. Admitted in the hospital for immunosuppressive treatments, with no clinical response, he developed worsening of inflammatory parameters, associated to decreased level of consciousness and hypotension responsive to fluids. ICU admission was requested for severe sepsis, with no need for artificial life support at this moment.

Patient 2. Male patient, 67 years-old, admitted to the hospital 39 days ago. Previous history of laryngeal carcinoma, tracheostomized and gastrostomized. Completely dependent for activities of daily living. Admitted to the hospital for clinical compensation from cachexia and dehydration, and treatment for aspirative pneumonia. He developed cardiac arrest due to hypoxemia during manipulation of the tracheostomy cannula. ICU admission was requested for post cardiac arrest status, in need for mechanical ventilation.

Considering the situation described above and the patients described on the vignettes, what is your decision regarding ICU admission of these patients?

a) Would admit patient 1 and refuse admission of patient 2
b) Would admit patient 2 and refuse admission of patient 1
c) Would refuse admission of both patients (1 and 2)
d) Would admit both patients (1 and 2)

D.2. Intensive care unit bed scarcity

You are the intensivist in charge of the intensive care unit (ICU) and is also responsible for decisions of ICU admission, when there is a request for the following patient. At the moment, there are only two available beds for admission, there is no other patient in conditions of discharge from the ICU and there are scheduled surgeries for the following day.

Patient 1. Female patient, 70 years-old, admitted to the hospital 22 days ago. Previous history of severe neurological sequelae due to stiff-person syndrome, refractory to treatments, already tracheostomized and with planned gastrostomy. Completely dependent for activities of daily living. Admitted in the hospital for immunosuppressive treatments, with no clinical response, he developed worsening of inflammatory parameters, associated to decreased level of consciousness and hypotension responsive to fluids. ICU admission was requested for severe sepsis, with no need for artificial life support at this moment.

Patient 2. Male patient, 67 years-old, admitted to the hospital 39 days ago. Previous history of laryngeal carcinoma, tracheostomized and gastrostomized. Completely dependent for activities of daily living. Admitted to the hospital for clinical compensation from cachexia and dehydration, and treatment for aspirative pneumonia. He developed cardiac arrest due to hypoxemia during manipulation of the tracheostomy cannula. ICU admission was requested for post cardiac arrest status, in need for mechanical ventilation.

Considering the situation described above and the patients described on the vignettes, what is your decision regarding ICU admission of these patients?

a) Would admit patient 1 and refuse admission of patient 2
b) Would admit patient 2 and refuse admission of patient 1
c) Would refuse admission of both patients (1 and 2)
d) Would admit both patients (1 and 2)

Group E - Single vignette not archetypical for intensive care unit admission or refusal

E.1. Non-intensive care unit bed scarcity

You are the intensivist in charge of the intensive care unit (ICU) and is also responsible for decisions for ICU admission, when there is a request for the following patient. At the moment, there are three available beds for admission and no other admission request.

Female patient, 16 years-old, admitted to the hospital hours ago. Previous history of splenectomy secondary to trauma accident 5 years ago and a cesarean delivery 21 days ago. Functionally independent for activities of daily living. She was admitted with a history of fever and abdominal pain, with a diagnosis of endometritis after complimentary evaluation. She is awake, not hypotensive, mild tachycardic with remaining vital signs normal, but has an altered arterial lactate level. Treatment was begun in the emergency room and ICU admission was requested for severe sepsis, without need for artificial life support at this moment.

Considering the situation described above and the patient described on the vignette, what is your decision regarding ICU admission of this patient?

a) Would admit the patient
b) Would refusal admission of the patient

E.2. Intensive care unit bed scarcity

You are the intensivist in charge of the intensive care unit (ICU) and is also responsible for decisions of ICU admission, when there is a request for the following patient. At the moment, there is only one available bed for admission, there is no other patient in conditions of discharge from the ICU and there are scheduled surgeries for the following day.

Female patient, 16 years-old, admitted to the hospital hours ago. Previous history of splenectomy secondary to trauma accident 5 years ago and a cesarean delivery 21 days ago. Functionally independent for activities of daily living. She was admitted with a history of fever and abdominal pain, with a diagnosis of endometritis after complimentary evaluation. She is awake, not hypotensive, mild tachycardic with remaining vital signs normal, but has an altered arterial lactate level. Treatment was begun in the emergency room and ICU admission was requested for severe sepsis, without need for artificial life support at this moment.

Considering the situation described above and the patient described on the vignette, what is your decision regarding ICU admission of this patient?

a) Would admit the patient
b) Would refusal admission of the patient

Group F - "Multiple-Choice" and "*Status Quo*" scenarios

F.1. "Multiple-choice" scenario

You are the intensivist in charge of the intensive care unit (ICU) and is also responsible for decisions of ICU admission, when there is a request for the following two patients. At the moment, there is only one available bed for admission, there is no other patient in conditions of discharge from the ICU and there are scheduled surgeries for the following day.

Patient 1. Male patient, 68 years-old, admitted to the hospital in the morning for an elective abdominal aortic aneurysm surgery. He is currently asymptomatic. Previous history of diabetes and hypertension. Functionally independent for activities of daily living. The surgery is scheduled to start in 6 hours. ICU admission was requested for post-operative monitoring of an elective abdominal aortic aneurysm surgery.

Patient 2. Male patient, 18 years-old, admitted to the hospital hours ago. No known past medical history. Functionally independent for activities of daily living. He was admitted to the emergency room following a motorcycle accident, presenting with severe traumatic brain injury (Glasgow coma score of 3). Head computerized tomography demonstrated pneumocranium, traumatic subarachnoid haemorrhage, brain swelling and complex face fractures, with no active surgical indication at the moment. No other injuries were found. ICU admission was requested for severe traumatic brain injury in need for monitoring and invasive mechanical ventilation.

Considering the situation described above and the patients described on the vignettes, what is your decision regarding ICU admission of these patients?

a) Would admit patient 1
b) Would admit patient 2
c) Would refuse admission of both patients 1 and 2

F.2. "*Status quo*" scenario

You are the intensivist in charge of the intensive care unit (ICU) and is also responsible for decisions of ICU admission, when there is a request for the following two patients. At the moment, there is only one available bed for admission, which is already reserved for a male patient, 68 years-old, asymptomatic, admitted electively for an abdominal aortic surgery scheduled to start in 6 hours. There is no other patient in conditions of discharge from the ICU and there are scheduled surgeries for the following day.

Patient 1. Female patient, 19 years, admitted to the hospital hours ago. No known previous medical history. Functionally independent for activities of daily living. Admitted to the emergency room following polytrauma secondary to being ran over by a car. She presented with cardiac arrest at the trauma scene, of short duration, reversed by the emergency medical system team and was intubated and placed on mechanical ventilation. Investigations demonstrated lung blunt trauma, and acute subdural haematoma with signs of brains swelling, with no active surgical indication at the moment. ICU admission was requested for severe traumatic brain injury associated to status post cardiopulmonary arrest, in need for invasive mechanical ventilation.

Patient 2. Male patient, 18 years-old, admitted to the hospital hours ago. No known past medical history. Functionally independent for activities of daily living. He was admitted to the emergency room following a motorcycle accident, presenting with severe traumatic brain injury (Glasgow coma score of 3). Head computerized tomography demonstrated pneumocranium, traumatic subarachnoid haemorrhage, brain swelling and complex face fractures, with no active surgical indication at the moment. No other injuries were found. ICU admission was requested for severe traumatic brain injury in need for monitoring and invasive mechanical ventilation.

Considering the situation described above and the patients described on the vignettes, what is your decision regarding ICU admission of these patients?

a) Would admit patient 1
b) Would admit patient 2
c) Would refuse admission of both patients 1 and 2

Table 1S - Number (proportion) of senior physicians that would admit each clinical vignette to the intensive care unit, stratified by intensive care unit bed scarcity or non-scarcity settings

	Admission to the ICU in a non-scarcity setting				Admission to the last ICU bed (ICU scarcity setting)	
	Completely agree	Agree	Disagree	Completely disagree	No	Yes
	n (%)	n (%)	n (%)	n (%)	n (%)	n (%)
Group A						
Patient	6 (75)	1 (13)	1(13)	0(0)	1(13)	7 (88)
Group B						
Patient 1	3 (38)	5 (63)	0 (0)	0 (0)	0 (0)	8 (100)
Patient 2	8 (100)	0 (0)	0 (0)	0 (0)	1 (13)	7 (88)
Group C						
Patient	0 (0)	2 (25)	4 (50)	2 (25)	8 (100)	0 (0)
Group D						
Patient 1	0 (0)	4 (50)	3 (38)	1 (13)	8 (100)	0 (0)
Patient 2	0 (0)	3 (38)	2 (25)	3 (38)	8 (100)	0 (0)
Group E						
Patient	3 (43)	3 (43)	1 (14)	0 (0)	4 (57)	3 (43)
Group F						
Multiple-Choice						
Patient 1 (dummy)						
Patient 2	3 (38)	5 (63)	0 (0)	0 (0)	0 (0)	8 (100)
Status quo						
Patient 1	5 (63)	3 (38)	0 (0)	0 (0)	1 (13)	7 (88)
Patient 2	3 (38)	5 (63)	0 (0)	0 (0)	0 (0)	8 (100)

ICU - intensive care unit.

Table 2S - Comparison of outcomes in respondents with complete and incomplete responses

Characteristics	Incomplete responses N (%)	Complete responses N (%)	p value
Group A vignettes			
Admitted	15 (100)	125 (100)	NA
Refused	0 (0)	0 (0)	
Perceived as difficult question	2 (13.3)	2 (1.6)	0.057
Group B vignettes			0.174
Admit patient 1 and refuse patient 2	1 (5.6)	0 (0)	
Admit patient 2 and refuse patient 1	0 (0)	2 (1.6)	
Both refused	0 (0)	1 (0.8)	
Both admitted	17 (94.4)	122 (97.6)	
Perceived as difficult question	0 (0)	5 (4.0)	0.388
Group C vignettes			0.265
Admitted	9 (56.3)	52 (41.6)	
Refused	7 (43.8)	73 (58.4)	
Perceived as difficult question	6 (37.5)	44 (35.2)	0.856
Group D vignettes			0.328
Admit patient 1 and refuse patient 2	1 (5.6)	8 (6.4)	
Admit patient 2 and refuse patient 1	1 (5.6)	25 (20.0)	
Both refused	11 (61.1)	51 (40.8)	
Both admitted	5 (27.8)	41 (32.8)	
Perceived as difficult question	7 (38.9)	56 (44.8)	0.637
Group E vignettes			0.267
Admitted	16 (100)	116 (92.8)	
Refused	0 (0)	9 (7.2)	
Perceived as difficult question	0 (0)	7 (5.6)	0.332
Group F vignettes			
Multiple-choice			0.840
Admit patient 1	1 (25.0)	9 (15.8)	
Admit patient 2	3 (75.0)	46 (80.7)	
Both refused	0 (0)	2 (3.5)	
Status quo			0.659
Admit patient 1	8 (88.9)	52 (76.5)	
Admit patient 2	1 (11.1)	13 (19.1)	
Both refused	0 (0)	3 (4.4)	
Perceived as difficult question	8 (61.5)	70 (56.0)	0.701

NA - not applicable.

Table 3S - Impact of distractors randomization on responses regarding intensive care unit allocation decisions

Characteristic	Control	Distractors	OR		95%CI	
	N (%)	N (%)	p value		Lower	Upper
Group A (single vignette archetypical for admission)						
Appropriate allocation (admitted)	68 (100)	57 (100)	NA	NA	NA	NA
Inappropriate allocation (refused)	0	0				
Perceived as difficult question *	0	2 (3.5)	0.119			
Group B (multiple vignettes archetypical for admission)			0.458	0.41	0.04	4.65
Appropriate allocation (both admitted)	67 (98.5)	55 (96.5)				
Inappropriate allocation (at least one refused)	1 (1.5)	2 (3.5)				
Perceived as difficult question **	3 (4.4)	2 (3.5)				
Group C (single vignette archetypical for refusal)			0.533	1.26	0.61	2.57
Appropriate allocation (refused)	38 (55.9)	35 (61.4)				
Inappropriate allocation (admitted)	30 (44.1)	22 (38.5)				
Perceived as difficult question¶	25 (36.8)	19 (33.3)	0.689			
Group D (multiple vignettes archetypical for refusal)			0.925	0.97	0.47	1.98
Appropriate allocation (both refused)	28 (41.2)	23 (40.4)				
Inappropriate allocation (at least one admitted)	40 (58.8)	34 (59.6)				
Perceived as difficult question†	26 (38.2)	30 (52.6)	0.107			
Group E (single non-archetypical vignette)			0.534	1.54	0.39	6.02
Admitted	64 (94.1)	52 (91.2)				
Refused	4 (5.9)	5 (8.8)				
Perceived as difficult question ‡	5 (7.4)	2 (3.5)	0.352			
Group F (cognitive biases vignettes)			0.175	2.28	0.68	7.72
Appropriate allocation	58 (85.3)	53 (93)				
Inappropriate allocation	10 (14.7)	4 (7)				
Perceived as difficult question	42 (61.8)	28 (49.1)	0.156			

OR - odds ratio. 95%CI - 95% confidence interval; NA - not applicable. * $p < 0.001$ in comparison to groups C, D and F; $p = 0.375$ in comparison to group B and $p = 0.18$ in comparison to group E. ** $p < 0.001$ in comparison to groups C, D and F; $p = 0.754$ in comparison to group E. ¶ $p < 0.001$ in comparison to groups E and F; $p = 0.05$ in comparison to group D. † $p < 0.001$ in comparison to group E; $p = 0.07$ in comparison to group F. ‡ $p < 0.001$ in comparison to group F.

Table 4S - Baseline characteristics accordingly to intensive care unit scarcity randomization

Characteristics	Group A (single vignette archetypical for admission)		p value	Group B (multiple vignettes archetypical for admission)		p value	Group C (single vignette archetypical for refusal)		p value
	ICU Availability (N = 55)	ICU Scarcity (N = 70)		ICU Availability (N = 57)	ICU Scarcity (N = 68)		ICU Availability (N = 56)	ICU Scarcity (N = 69)	
Time to complete questionnaire (minutes)	20.4 ± 27.7	20.5 ± 26.5	0.985	20.2 ± 28.5	20.7 ± 25.7	0.916	18.9 ± 26.3	21.7 ± 27.5	0.575
Age	36.4 ± 6.6	38.2 ± 7.8	0.169	38.2 ± 8.1	36.7 ± 6.6	0.271	36.9 ± 7.0	37.8 ± 7.6	0.503
Male sex	41 (74.5)	46 (66.7)	0.341	39 (68.4)	48 (71.6)	0.696	36 (64.3)	51 (75.0)	0.194
Years of medical practice	12.0 ± 6.9	13.6 ± 8.5	0.253	13.9 ± 8.6	12.1 ± 7.1	0.201	12.3 ± 7.7	13.4 ± 7.9	0.439
Board certified in critical care	43 (78.2)	52 (74.3)	0.613	42 (73.7)	53 (77.9)	0.579	40 (71.4)	55 (79.7)	0.281
Average hours working in ICU per week (hours)			0.052			0.648			0.150
< 12	2 (3.6)	2 (2.9)		3 (5.3)	1 (1.5)		0 (0)	4 (5.8)	
12 - 24	2 (3.6)	13 (18.6)		7 (12.3)	8 (11.8)		9 (16.1)	6 (8.7)	
24 – 40	20 (36.4)	16 (22.9)		17 (29.8)	19 (27.9)		14 (25.0)	22 (31.9)	
> 40	31 (56.4)	39 (55.7)		30 (52.6)	40 (58.8)		33 (58.9)	37 (53.6)	
"Closed" ICU	35 (63.6)	42 (60.0)	0.678	34 (59.6)	43 (63.2)	0.681	33 (58.9)	44 (63.8)	0.586
Public ICU	28 (50.9)	30 (42.9)	0.370	28 (49.1)	30 (44.1)	0.576	22 (39.3)	36 (52.2)	0.151
High-intensity staff ICU	53 (96.4)	70 (100)	0.108	56 (98.2)	67 (98.5)	0.900	55 (98.2)	68 (98.6)	
Number of ICU beds	23.4 ± 16.8	21.6 ± 15.6	0.523	23.9 ± 17.2	21.2 ± 15.2	0.353	23.0 ± 16.4	21.9 ± 16.0	0.697

Continue...

...Continuation

Experience of situations of ICU beds scarcity			0.641			0.992			0.941
Never	4 (7.3)	2 (2.9)		3 (5.3)	3 (4.4)		2 (3.6)	4 (5.8)	
Rarely	12 (21.8)	20 (28.6)		14 (24.6)	18 (26.5)		16 (28.6)	16 (23.2)	
Sometimes	13 (23.6)	20 (28.6)		16 (28.1)	17 (25.0)		14 (25.0)	19 (27.5)	
Frequently	14 (25.5)	16 (22.9)		13 (22.8)	17 (25.0)		13 (23.2)	17 (24.6)	
Always	12 (21.8)	12 (17.1)		11 (19.3)	13 (19.1)		11 (19.6)	13 (18.8)	
Involved in ICU triage			0.377			0.796			0.734
Never	15 (27.3)	16 (22.9)		14 (24.6)	17 (25.0)		17 (30.4)	14 (20.3)	
Rarely	5 (9.1)	16 (22.9)		10 (17.5)	11 (16.2)		8 (14.3)	13 (18.8)	
Sometimes	12 (21.8)	13 (18.6)		12 (21.1)	13 (19.1)		11 (19.6)	14 (20.3)	
Frequently	15 (27.3)	17 (24.3)		16 (28.1)	16 (23.5)		14 (25.0)	18 (26.1)	
Always	8 (14.5)	8 (11.4)		5 (8.8)	11 (16.2)		6 (10.7)	10 (14.5)	
Previous training in ICU triage	12 (21.8)	8 (11.4)	0.116	9 (15.8)	11 (16.2)	0.953	11 (19.6)	9 (13.0)	0.317
Perceived difficult in answering the complete questionnaire	2.5 (1.5 - 3.0)	2.0 (2.0 - 3.0)	0.784	2.0 (2.0 - 3.0)	2.5 (2.0 - 3.0)	0.435	2.25 (2.0 - 3.0)	2.5 (2.0 - 3.0)	0.949

Characteristics	Group D (multiple vignettes archetypical for refusal)		p value	Group E (single non-archetypical vignette)		p value
	ICU Availability (N=56)	ICU Scarcity (N=69)		ICU Availability (N=61)	ICU Scarcity (N=64)	
Time to complete questionnaire (minutes)						
15.7 ± 11.8	24.4 ± 34.3	0.073	19.7 ± 31.6	21.2 ± 21.7	0.752	
Age	38.2 ± 8.5	36.7 ± 6.3	0.288	37.0 ± 7.5	37.7 ± 7.3	0.587
Male sex	40 (72.7)	47 (68.1)	0.577	44 (72.1)	43 (68.3)	0.637
Years of medical practice	13.7 ± 9.1	12.3 ± 6.7	0.305	12.5 ± 8.2	13.3 ± 7.6	0.577
Board certified in critical care	40 (71.4)	55 (79.7)	0.281	43 (70.5)	52 (81.3)	0.159
Average hours working in ICU per week (hours)			0.088			0.874
< 12	4 (7.1)	0 (0)		2 (3.3)	2 (3.1)	
12 - 24	8 (14.3)	7 (10.1)		6 (9.8)	9 (14.1)	
24 – 40	17 (30.4)	19 (27.5)		19 (31.1)	17 (26.6)	
> 40	27 (48.2)	43 (62.3)		34 (55.7)	36 (56.3)	
"Closed" ICU	38 (67.9)	39 (56.5)	0.195	38 (62.3)	39 (60.9)	0.876
Public ICU	31 (55.4)	27 (39.1)	0.070	27 (44.3)	31 (48.4)	0.640
High-intensity staff ICU	55 (98.2)	68 (98.6)	0.881	61 (100)	62 (96.9)	0.164
Number of ICU beds	21.3 ± 14.5	23.3 ± 17.4	0.507	22.8 ± 16.4	22.0 ± 16.0	0.782
Experience of situations of ICU beds scarcity			0.408			0.737
Never	2 (3.6)	4 (5.8)		4 (6.6)	2 (3.1)	
Rarely	12 (21.4)	20 (29.0)		16 (26.2)	16 (25.0)	
Sometimes	14 (25.0)	19 (27.5)		16 (26.2)	17 (26.6)	
Frequently	18 (32.1)	12 (17.4)		12 (19.7)	18 (28.1)	
Always	10 (17.9)	14 (20.3)		13 (21.3)	11 (17.2)	
Involved in ICU triage			0.131			0.847
Never	11 (19.6)	20 (29.0)		16 (26.2)	15 (23.4)	
Rarely	12 (21.4)	9 (13.0)		11 (18.0)	10 (15.6)	
Sometimes	9 (16.1)	16 (23.2)		10 (16.4)	15 (23.4)	
Frequently	19 (33.9)	13 (18.8)		15 (24.6)	17 (26.6)	
Always	5 (8.9)	11 (15.9)		9 (14.8)	7 (10.9)	
Previous training in ICU triage	7 (12.5)	13 (18.8)	0.336	13 (21.3)	7 (10.9)	0.114
Perceived difficult in answering the complete questionnaire	2.5 (2.0 - 3.0)	2.0 (2.0 - 3.0)	0.717	2.0 (1.5 - 2.5)	2.5 (2.0 - 3.0)	0.057

ICU – intensive care unit. Results expressed at mean ± standard deviation, n (%) or median (interquartile range).

Table 5S - Impact of intensive care unit scarcity randomization on responses regarding intensive care unit allocation decisions

Characteristics	ICU availability	ICU scarcity	p value	OR	95%CI	
	N (%)	N (%)			Lower	Upper
Group A (single vignette archetypical for admission)						
Appropriate allocation (admitted)	55 (100)	70 (100)	NA	NA	NA	NA
Inappropriate allocation (refused)	0	0				
Perceived as difficult question*	1 (1.8)	1 (1.4)	0.863			
Group B (multiple vignettes archetypical for admission)						
Appropriate allocation (both admitted)	56 (98.2)	66 (97.1)	0.666	0.59	0.05	6.67
Inappropriate allocation (at least one refused)	1 (1.8)	2 (2.9)				
Perceived as difficult question **	0 (0)	5 (7.4)	0.037			
Group C (single vignette archetypical for refusal)						
Appropriate allocation (refused)	26 (46.4)	47 (68.1)	0.014	2.47	1.19	5.11
Inappropriate allocation (admitted)	30 (53.6)	22 (31.9)				
Perceived as difficult question¶	17 (30.4)	27 (39.1)	0.307			
Group D (multiple vignettes archetypical for refusal)						
Appropriate allocation (both refused)	22 (39.3)	29 (42.0)	0.756	1.12	0.55	2.30
Inappropriate allocation (at least one admitted)	34 (60.7)	40 (58.0)				
Perceived as difficult question	23 (41.1)	33 (47.8)	0.45			
Group E (single non-archetypical vignette)						
Admitted	59 (96.7)	57 (89.1)	0.098	3.62	0.72	18.18
Refused	2 (3.3)	7 (10.9)				
Perceived as difficult question	0 (0)	7 (10.9)	0.008			

ICU - intensive care unit; OR - odds ratio; 95%CI - 95% confidence interval; NA - not applicable. **$p < 0.001$ in comparison to groups C, D and F; $p = 0.754$ in comparison to group E. ¶$p < 0.001$ in comparison to groups E and F; $p = 0.05$ in comparison to group D

Table 6S- Baseline characteristics accordingly to multiple-choice/*status quo* randomization

Characteristics	Group F (cognitive biases vignettes)		p value
	Multiple-choice (N = 57)	*Status quo* (N = 68)	
Time to complete questionnaire (minutes)	19.0 ± 18.3	21.7 ± 32.5	0.583
Age	36.9 ± 8.1	37.8 ± 6.6	0.529
Male sex	43 (75.4)	44 (65.7)	0.236
Years of medical practice	12.3 ± 8.7	13.5 ± 7.1	0.414
Board certified in critical care	43 (75.4)	52 (76.5)	0.893
Average hours working in ICU per week			0.72
< 12 hours	1 (1.8)	3 (4.4)	
12 - 24 hours	8 (14.0)	7 (10.3)	
24 - 40 hours	15 (26.3)	21 (30.9)	
> 40 hours	33 (57.9)	37 (54.4)	
"Closed" ICU	34 (59.6)	43 (63.2)	
Public ICU	27 (47.4)	31 (45.6)	0.842
High-intensity staff ICU	56 (98.2)	67 (98.5)	0.9
Number of ICU beds	23.3 ± 16.7	21.6 ± 15.8	0.559
Experience of situations of ICU beds scarcity			0.911
Never	2 (3.5)	4 (5.9)	
Rarely	13 (22.8)	19 (27.9)	
Sometimes	16 (28.1)	17 (25.0)	
Frequently	14 (24.6)	16 (23.5)	
Always	12 (21.1)	12 (17.6)	
Involved in ICU triage			
Never	15 (26.3)	16 (23.5)	0.627
Rarely	12 (21.1)	9 (13.2)	
Sometimes	9 (15.8)	16 (23.5)	
Frequently	15 (26.3)	17 (25.0)	
Always	6 (10.5)	10 (14.7)	
Previous training in ICU triage	7 (12.3)	13 (19.1)	0.299
Perceived difficult in answering the complete questionnaire	2.0 (1.5 - 2.5)	2.5 (2.0 - 3.0)	0.052

ICU - intensive care unit. Results expressed at mean ± standard deviation, n (%) or median (interquartile range).

Table 7S - Impact of multiple-choice/*status quo* randomization on responses regarding intensive care unit allocation decisions

Characteristics	Multiple-choice	*Status quo*	OR		95%CI	
	N (%)	N (%)	p value		Lower	Upper
Group F (cognitive biases vignettes)						
Appropriate allocation	46 (80.7)	65 (95.6)	0.009	5.18	1.37	19.62
Inappropriate allocation	11 (19.3)	3 (4.4)				
Perceived as difficult question	22 (38.6)	48 (70.6)	< 0.001			

OR - odds ratio; 95%CI - 95% confidence interval.

Table 8S - Number of patients in each interaction group

	Control	Cognitive load	Total
Group A			
Control	31	24	55
ICU scarcity	37	33	70
Total	68	57	125
Group B			
Control	30	27	57
ICU scarcity	38	30	68
Total	68	57	125
Group C			
Control	27	29	56
ICU scarcity	41	28	69
Total	68	57	125
Group D			
Control	30	26	56
ICU scarcity	38	31	69
Total	68	57	125
Group E			
Control	31	30	61
ICU scarcity	37	27	64
Total	68	57	125
Group F			
Multiple-choice	31	26	57
Status quo	37	31	68
Total	68	57	125

ICU - intensive care unit.
